# Understanding protective and risk factors affecting adolescents’ well-being during the COVID-19 pandemic

**DOI:** 10.1038/s41539-022-00149-4

**Published:** 2022-12-14

**Authors:** Min Lan, Qianqian Pan, Cheng Yong Tan, Nancy Wai Ying Law

**Affiliations:** 1 Key Laboratory of Intelligent Education Technology and Application of Zhejiang Province, Zhejiang Normal University, Jinhua, China; 2 Centre for Research in Pedagogy and Practice (CRPP), Office of Education Research, National Institute of Education, Nanyang Technological University, Singapore, Singapore; 3 Faculty of Education, The University of Hong Kong, Hong Kong, China

**Keywords:** Education, Human behaviour

## Abstract

This study investigated the factors affecting adolescents’ well-being during the COVID-19 pandemic from the perspectives of their participation in digital activities, emotional regulation, self-regulated learning, and parental involvement. Using self-reported data from 932 pairs of adolescents and their parents, we performed multiple-group structural equation modeling, which revealed that self-efficacy in online learning during school suspension was a key factor influencing adolescents’ perceived worries after schools resumed. During school suspension, boys’ cognitive-emotional regulation played a protective role in their well-being, helping them to avoid cyberbullying incidents, while girls’ participation in leisure-oriented digital activities compromised their self-efficacy in online learning and led to cyberbullying incidents. Furthermore, improvement in parent–child relationships during school suspension encouraged adolescents to use more positive emotional regulation strategies, enhanced their self-efficacy in online learning, and reduced their leisure-time digital activities. The findings indicate that the effective regulation of adolescents’ online behaviors, emotions, and self-efficacy, especially when combined with an emotionally secure family relationship, can ensure adolescents’ well-being.

## Introduction

Because of COVID-19-related school suspensions, adolescents in Hong Kong lacked the normal face-to-face schooling and socialization for a prolonged period. Instead, the government policy of “suspending classes without suspending learning” meant that students engaged in more online learning and socialization^1^. The effect of such changes on adolescents’ studying and their daily life deserves our attention because their experiences (e.g., negative affection^2^) during school suspensions may influence their well-being after schools resumed^3^.

**Fig. 1:**
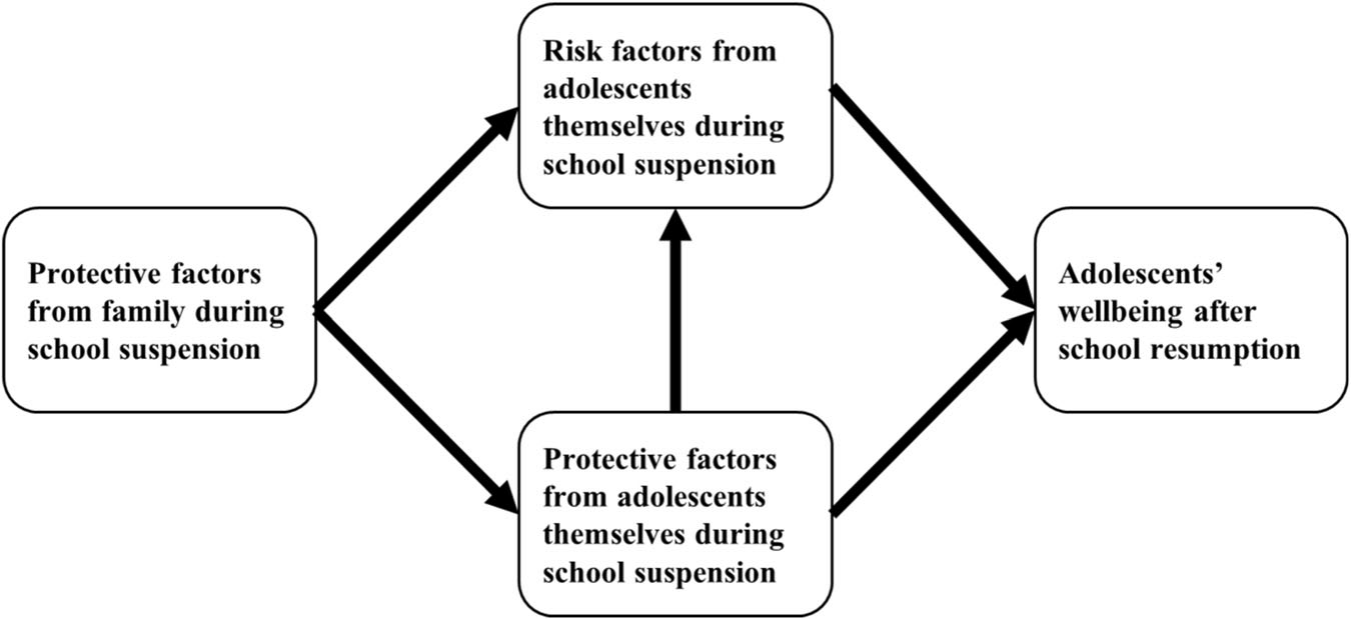
Conceptual framework of protective and risk factors during school suspension for adolescents' well-being after school resumption. Protective factors from family during school suspension were supposed to be associated with both risk and protective factors from adolescents themselves. Adolescents' protective factors were assumed to be associated with their risk factors. Both risk and protective factors were directly associated with adolescents' well being after school resumption.

To cope with the challenges of online study or non-study activities, aspects of adolescents’ ability to self-regulate during school suspension were viewed as direct or indirect factors that would protect their well-being after schools reopened^3^. Moreover, from the perspective of the family, parental support was also important in maintaining their children’s well-being. In this study, we investigated the association between adolescents’ well-being and their self-regulation in terms of behavior, emotions, and cognition, and their parents’ support (see Fig. 1 for the conceptual framework).

Greater engagement in digital activities while schools were suspended during the pandemic brought with it potential risks, difficulties, and conflicts. For instance, more participation in digital activities for entertainment (e.g., online gaming or browsing social media) was associated with increased psychological distress and anxiety in adolescents^1^. In addition, negative experiences from digital-social activities may have increased their exposure to cyberbullying^4,5^ (e.g., sending or receiving hostile or aggressive messages intended to inflict harm or discomfort on each other^6^), which may have further impaired their psychological well-being^7^. Furthermore, with more time spent on digital socialization, adolescents had less time for study, which could have resulted in lower academic self-efficacy^8^. As lower learning self-efficacy may influence adolescents’ learning performance, school suspension may have increased their worries about their learning outcomes and daily life after schools resumed^7,9^.

In the face of negative events during school suspension, whether online or face-to-face, it is important for adolescents to be able to use appropriate self-regulatory strategies to cope with the influx of emotionally charged information. Emotional regulation, which is defined as the “*conscious, mental strategies individuals use to handle the intake of emotionally arousing information*”^10^, is an important self-regulatory process developed in adolescence when individuals learn to cope with emotional experiences arising from negative events^11^.

Emotional regulation strategies can protect against emotional problems^12^ and enhance well-being^13^. For instance, the capacity for self-regulatory resilience and positivity plays a crucial protective role in ensuring emotional and cognitive well-being after involvement in cyberbullying incidents^14,15^. For academic study, emotional regulation may modulate the effects of emotional states on adolescents’ academic self-efficacy^16^.

Increased opportunities for interaction between parents and children during the COVID-19 school suspension may have improved their relationships^17^ but also cause more family conflicts. To understand how adolescents’ well-being was affected during school suspension, family factors cannot be ignored. According to spillover theory^18^, individuals are embedded in various interdependent social systems. Changes in one system (e.g., family relationships) can alter the correlates (e.g., emotions) that can affect social interactions in other systems (e.g., peer relationships).

Improvement in parent–child relationships and parents’ appropriate monitoring may have benefited their children’s self-regulation^19^. A healthy parent–child relationship and parental monitoring have both been found to be associated with improving adolescents’ emotional regulation strategies^20,21^ and helping to maintain their social-emotional wellness in other connected systems. For instance, in seeking to ameliorate the feelings of emptiness and loneliness caused by a poor parent–child relationship^22^, adolescents may become addicted to online socialization as they share and communicate their feelings with others^23^.

Different types of parental monitoring such as restrictive^21^ or neglectful styles^23^ can affect adolescents’ participation in both academic and non-academic digital activities. For instance, a good parent–child relationship and school-related parental monitoring can have positive effects on children’s academic self-efficacy^24,25^. In contrast, ineffective parental monitoring may result in children’s involvement in cyberbullying incidents^26–28^.

Sex differences have been shown to influence both adolescents’ individual factors (e.g., adolescents’ participation in digital activities^29,30^, involvement^15^ in cyberbullying^31,32^, academic self-efficacy^33^, psychological status^34,35^, use of cognitive emotional regulation strategies^36,37^) and family factors (e.g., parental involvement^38,39^). Sex differences also exist in the interactions of these factors^15,29,39,40^. From a developmental perspective, adolescents respond to regulations on emotions^36,41^, behaviors^42^, and cognition^43^ differently as their age increases.

As self-regulatory processes can vary across environments, the conditions during the COVID-19 pandemic can provide new insights into how adolescents perceive, think, and behave during epidemiological disasters, as well as the effects on their daily life and their studies after the pandemic. This study aimed to understand the effect of adolescents’ individual and family factors on online and offline social-ecological systems during school suspensions and the links of those systems to adolescents’ well-being after school resumption.

First, studies^1,4,5^ have shown that it is unclear whether and to what extent participation in digital-social activities, particularly cyberbullying incidents (i.e., negative experiences), were associated with adolescents’ self-regulation in their online studies and emotions during the COVID-19-related school suspension and their well-being after school resumption. The present study presents us with an opportunity to investigate these associations, which can help protect adolescents’ well-being in the future when facing the changes and challenges of daily life and study during difficult periods.

Second, we do not know whether adolescents’ self-regulatory behaviors, emotions, and cognition can be influenced or protected by the quality of parent–child relationships and parental monitoring^27,44–46^, particularly with parents and children spending more time together during COVID-19 lockdowns^17^. From the perspective of parental involvement, the COVID-19-related school suspension has provided us with a natural setting to investigate whether and how these changes benefit or impede children’s self-regulatory processes toward changes or challenges in the future.

In addition, the findings on sex and age differences within such factors and associations are inconclusive^47^. Sex and age perspectives must be taken into account to provide insights into how best to support adolescents in future. This study conducted a multiple group analysis to investigate the above associations in relation to sex and age differences.

The results of this exploration can provide insights for parents, teachers, and school leaders from the perspectives of both the family and the school to equip adolescents with the self-regulatory capacity they need when facing similar challenges in the future.

## Results

Self-reported data were obtained from the “eCitizen Education 360” project, which explored how to help students adjust to their studies and daily life in the New Normal brought about by the pandemic. A total of 932 students from 23 secondary schools in Hong Kong completed surveys on cognitive-emotional regulation strategies (CER), participation in digital activities for socialization and entertainment (DSE), online learning self-efficacy (OSE), cyberbullying involvement (CyI), and their perceived worries for future study and life (WOR). Their parents completed surveys on the perceived improvement in the parent–child relationship (PCR) and parental monitoring (PM) of their children’s online activities.

### The goodness of the model fit

The goodness of the model fit of all the confirmatory factor analysis (CFA) models was satisfactory, which was supported by a comparative fit index (CFI) above .95 and root mean square error of approximation (RMSEA) below .08 (see Table 1). The model fit for cyberbullying involvement was examined by reviewing the residual plots for various ability subgroups, which sufficiently supported the model fit. The reliability for each scale was satisfied with values of Ω_CER_ = 0.71, Ω_DSE_ = 0.74, Ω_OSE_ = 0.82, Ω_WOR_ = 0.73, Ω_PCR_ = 0.84, and Ω_PM_ = 0.74 and empirical reliability of CyI = 0.79. Detailed psychometric analysis procedures, the results of the model fit, and the empirical reliability of CyI, can be found in the method section. This study performed multiple-group structural equation modeling (MG-SEM) for female and male students based on these factors. The model fit of MG-SEM was satisfactory (RMSEA = 0.048, CFI = 0.903, standardized root mean squared residual (SRMR) = 0.065).

**Table 1:** Results of construct validity and reliability.

Variables	Items	Factor loadings (SE)	CFI	RMSEA	Reliability (Ω)
Student variables					
Digital activities participation for socialization and entertainment (DSE)	Item 1	0.63** (0.04)	1.00	0.04	0.74
	Item 2	0.64** (0.03)			
	Item 3	0.72** (0.05)			
	Item 4	0.41** (0.03)			
	Item 5	0.52** (0.03)			
Cognitive-emotional regulation strategies (CER)	Item 1	0.45** (0.05)	1.00	0.00	0.71
	Item 2	0.69** (.05)			
	Item 3	0.85** (0.04)			
Online learning self-efficacy (OSE)	Item 1	0.55** (0.03)	0.98	0.07	0.82
	Item 2	0.74** (0.03)			
	Item 3	0.52** (0.03)			
	Item 4	0.81** (0.02)			
	Item 5	0.79** (0.02)			
	Item 6	0.57** (0.03)			
Perceived worries for future study and life (WOR)	Item 1	0.65** (0.03)	0.97	0.06	0.73
	Item 2	0.75** (0.03)			
	Item 3	0.62** (0.03)			
	Item 4	0.49** (0.03)			
	Item 5	0.51** (0.03)			
	Item 6	0.50** (0.04)			
Parent variables					
Parental monitoring of their children’s online activities (PM)	Item 1	0.72** (0.02)	1.00	0.00	0.72
	Item 2	0.96** (0.01)			
	Item 3	0.86** (0.02)			
Improvement of parent-child relationship (PCR)	Item 1	0.81** (0.02)	1.00	0.01	0.84
	Item 2	.77** (0.03)			
	Item 3	0.78** (0.02)			
	Item 4	0.76** (0.03)			

### Adolescents’ participation in digital activity during school suspension and their well-being

**Fig. 2:**
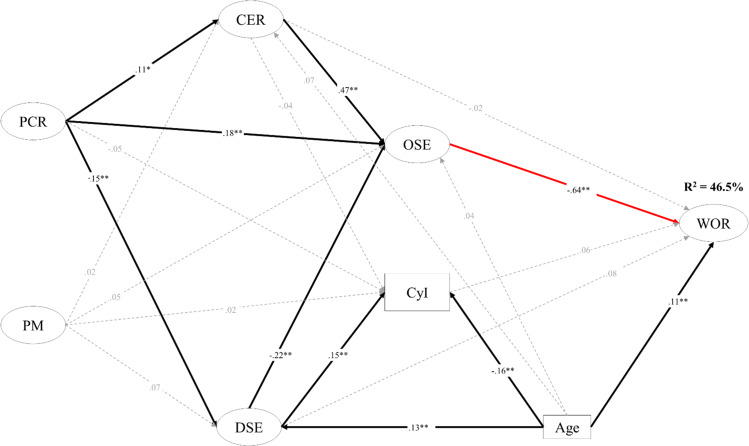
Results of the multiple-group structural equation modeling (Female group). PCR Parent-child relationship improvement, PM Parental monitoring, CER Cognitive-emotional regulation,; DSE Digital activities for socialization and entertainment, OSE Online learning self-efficacy, CyI Cyberbullying involvement, WOR Perceived worries for future study and life. Black arrows represent statistically significant direct effect, dotted arrows represent statistically insignificant direct effect, and red arrow represents statistically significant different direct effect between male group and female group. *p < 0.05. **p < 0.01.

**Fig. 3:**
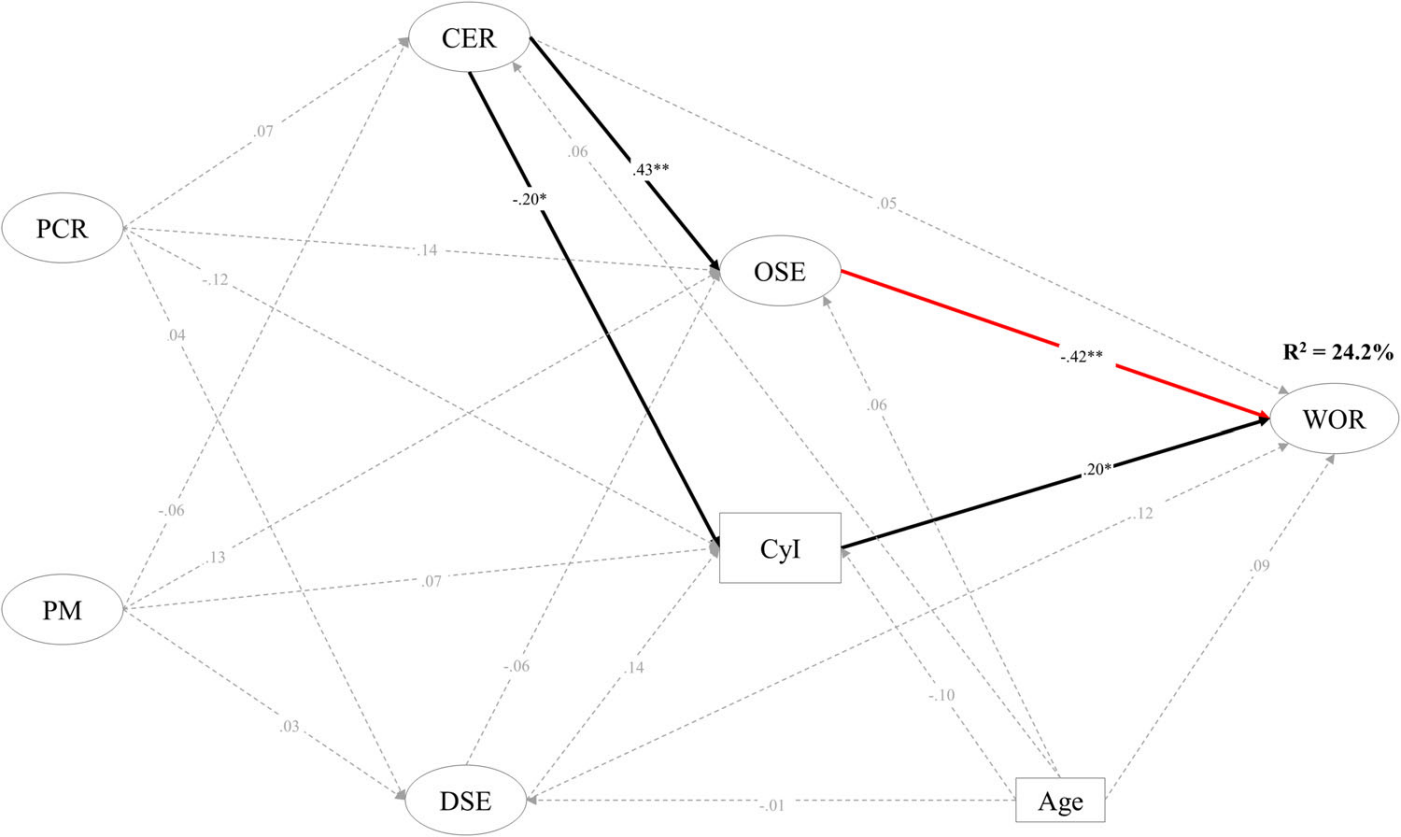
Results of the multiple-group structural equation modeling (Male group). PCR Parent-child relationship improvement, PM Parental monitoring, CER Cognitive-emotional regulation, DSE Digital activities for socialization and entertainment, OSE Online learning self-efficacy, CyI Cyberbullying involvement, WOR Perceived worries for future study and life. Black arrows represent statistically significant direct effect, dotted arrows represent statistically insignificant direct effect, and red arrow represents statistically significant different direct effect between male group and female group. *p < 0.05. **p < 0.01.

The participation of both girls (see Fig. 2) and boys (see Fig. 3) in DSE during the COVID-19-related school suspension was not significantly associated with adolescents’ perceived worries for study and life (WOR) after school resumed.

Girls’ DSE was negatively associated with OSE (β = −0.22, *p* < 0.01) but positively associated with CyI (β = 0.15, *p* < 0.01). Boys’ DSE was not significantly associated with any other variable (i.e., OSE, CyI, and WOR).

CyI was positively associated with WOR for boys (β = 0.20, *p* < 0.05), while there was no significant association between CyI and WOR for girls (β = 0.06, *p* = 0.17).

### Adolescents’ self-regulation during school suspension and their well-being

Use of CER during school suspension was positively associated with adolescents’ OSE (β_*girls*_ = 0.47, *p* < 0.01; β_*boys*_ = 0.43, *p* < 0.01). OSE was significantly associated with perceived WOR both for girls (β = −0.64, *p* < 0.01) and boys (β = −0.42, *p* < 0.01).

The association between OSE during school suspension and their WOR after school resumption for girls was significantly stronger (D_OSE→WOR_ = 0.35, *p* < 0.05) than for boys, controlling for student age. CyI was significantly associated with CER for boys (β = −0.20, *p* < .05) but not for girls (β = −0.04, *p* = 0.43).

### Effects of adolescents’ age and overall effects of digital activity and self-regulation factors

Age was not significantly associated with the variables of DSE, CyI, CER, and OSE during school suspension for boys. These four variables together explained 24.2% of the variance in boys’ WOR after school resumption.

Girls’ age was significantly associated with their DSE (β = 0.13, *p* < 0.01) and CyI (β = −0.16, *p* < 0.01) during school suspension, and with WOR (β = 0.11, *p* < 0.01) after school resumption. The variables DSE, CyI, CER, and OSE during school suspension explained 46.5% of the variance in WOR for girls after school suspension.

### Family factors in adolescents’ digital activity participation and self-regulation during school suspension

During the school suspension, girls’ relationship with their parents (PCR) was positively associated with their use of CER (β = .11, *p* < .05) and OSE (β = 0.18, *p* < 0.01) and negatively associated with their DSE (β = −0.15, *p* < 0.01) but was not significantly associated with CyI (β = −0.05, *p* = 0.29). Girls’ PM was not significantly associated with any of the digital activity and self-regulation factors (i.e., CER, OSE, CyI, and DSE). For boys, neither their PCR nor PM was significantly associated with any of these four factors.

### Mediator effects of adolescents’ digital activities participation and self-regulation on their well-being

Using CER and the improvement in girls’ PCR during school suspension were both negatively associated with their WOR after school resumed through their OSE during school suspension (CER: β = −0.35, *p* < 0.01; PCR: β = −0.11, *p* < 0.01), whereas the DSE of girls was positively associated with WOR through OSE (β = 0.13, *p* < 0.01).

The PCR of girls during school suspension was negatively associated with WOR through CER and OSE during school suspension in sequence (β = −0.03, *p* < 0.05) and through DSE and OSE during school suspension in sequence (β = −0.02, *p* < 0.05).

Boys’ CER during school suspension was negatively associated with WOR after school resumption and through OSE during school suspension (β = −.19, *p* < .01).

The indirect effects from CER to WOR through OSE (D_CER → OSE → WOR_ = 0.16, *p* < 0.05, see Table 2), DSE to WOR through OSE (D_DES → OSE → WOR_ = −0.11, *p* < 0.05), and PCR to WOR through DSE and OSE (D _PCR → DES → OSE → WOR_ = 0.02, *p* < 0.05) were significantly stronger in female adolescents than in males.

**Table 2:** Results of indirect effects and group comparisons.

	Male		Female		Difference	
Path	Coefficient	p-value	Coefficient	p-value	Coefficient	*p*-value
CER→OSE→WOR	**−0.19**	**	**−0.35**	**	0.16	*
CER→CyI → WOR	−0.04		0.00		−0.04	
DSE→OSE→WOR	0.02		**0.13**	**	−0.11	*
DSE→CyI → WOR	0.02		0.01		0.02	
PCR→OSE→WOR	−0.05		**−0.11**	**	0.06	
PCR→CER→WOR	0.00		0.00		0.01	
PCR→CyI → WOR	−0.02		0.00		−0.02	
PCR→CER→OSE→WOR	−0.01		**−0.03**	*	0.02	
PCR→CER→CyI → WOR	0.00		0.00		0.00	
PCR→DSE→OSE→WOR	0.00		**−0.02**	*	0.02	*
PCR→DSE→CyI → WOR	0.00		0.00		0.00	
PM→CER→WOR	0.00		0.00		0.00	
PM→OSE→WOR	−0.03		−0.02		−0.01	
PM→CyI → WOR	0.01		0.00		0.01	
PM→CER→OSE→WOR	0.01		0.00		0.01	
PM→CER→CyI → WOR	0.00		0.00		0.00	
PM→DSE→OSE→WOR	0.00		0.00		−0.01	
PM→DSE→CyI → WOR	0.00		0.00		0.00	

*PCR* Parent-child relationship improvement, *PM* Parental monitoring, *CER* Cognitive-emotional regulation, *DSE* Digital activities for socialization and entertainment; *OSE* Online learning self-efficacy, *CyI* Cyberbullying involvement, *WOR* Perceived worries for future study and life.

*p < 0.05. **p < 0.01.

## Discussion

This study demonstrated that adolescents’ well-being after school resumed was affected by risk and protective factors from the adolescents themselves and by protective factors from their families during the COVID-19 school suspension. As a potential risk factor, adolescents engaging in more DSE during school suspension was not a direct risk factor affecting their perceived WOR after school resumption, as is usually assumed. Although engaging in more DSE during school suspension may have potentially increased girls’ CyI, the increase did not appear to influence their WOR after school resumption. In contrast, boys who engaged in more DSE during the period did not increase their CyI, but their CyI may have negatively influenced their WOR after school resumption.

As a protective factor, higher OSE during school suspension may have protected adolescents by reducing their WOR after school resumption. However, the protective effect was higher for girls, although girls’ DSE engagement may have been a risk factor that lowered their OSE during school suspension. As another protective factor, adolescents’ use of more CER when faced with negative events during school suspension was positively associated with their OSE. For boys, CER even protected them from CyI during school suspension.

A supportive family environment (i.e., PCR improvement) during school suspension helped safeguard girls’ behavioral and emotional well-being, leading to less DSE involvement, more CER use, and improvement in OSE. Furthermore, as a protective factor, PCR was also helpful in strengthening girls’ OSE during school suspension and reducing their WOR after school resumption. However, this protective effect was not found in the boys group.

As highlighted by Zimmerman when writing about the importance of self-regulation^48^, adolescents’ OSE (i.e., their motivational beliefs about self-learning) during school suspension was found to have a large influence over their WOR after school resumed. In particular, the effect of OSE on these worries was much stronger in female adolescents. Yeo et al.^47^ explained that girls were developmentally more likely to develop a greater sense of personal responsibility for negative events in general. In another study of Hong Kong students, Hui indicated that female adolescents made more internal attributions—focusing on one’s own abilities and efforts—and shouldered more personal responsibility for their concerns^49^. As studying went online, students needed to perform more self-directed learning, and girls’ regulatory capability may have been stimulated more at this stage of development. Furthermore, the current study demonstrated that OSE explains about double the variance in adolescents’ WOR after school resumption. This may be because female adolescents exhibited higher academic self-regulation—greater ability to focus their attention and to monitor themselves, and more self-control^42^—in an online learning context at home, which left them well-prepared for the change from school to home study.

As shown in a recent study^50^, the use of positive CER to deal with COVID-19-related experiences may have a positive association with adolescents’ beliefs in their online learning capabilities during school suspension, especially in female adolescents. This process of positive emotional regulation plays a role in buffering adolescents’ intense moods that might otherwise lead to affective influences on cognition^16^, thereby protecting them against emotional and academic exhaustion^50,51^.

Positive CER protected male adolescents from involvement in CyI during the school suspension and reduced their worries about daily life after the school resumption. According to Gianesini and Brighi^15^, various emotional regulation skills in cyberbullying victims or perpetrators could greatly influence adolescents’ resilience levels, which may further cushion them from the negative influences of peer violence during adolescence.

Echoing research^1,5^ showing the negative influence on adolescents’ studies and daily life of participation in digital activities, this study indicated that DSE resulted in adolescents showing lower OSE during school suspension and probably resulted in a greater involvement in CyI, and that girls are probably more vulnerable than boys to negative influences from the Internet^52^. From the cognitive perspective, brain structure and development in males and females may be different, with a greater influence on the regions related to cognitive control and reward/loss processing in females^52^. However, from a cultural perspective, this is probably because Chinese parents traditionally allocate more resources and guidance to their sons than to their daughters^53^. Parents monitor their sons and stop them from over-engaging in leisure-oriented digital activities, instead encouraging them to gain digital skills for studying.

However, as suggested in a study by Barlett et al.^4^, which did not consider the issue of age, this study showed that cyberbullying issues decreased with age for girls but not for boys. According to the Pew Research Center^54^, young girls experience particularly severe forms of online harassment. However, girls might develop self-regulatory digital resilience^14,15^ (e.g., understanding more about different types of cyberbullying and knowing how to handle them) earlier than boys to reduce their CyI. For boys, DSE was insignificantly associated with CyI, indicating that there might be factors in their other offline social systems^18^ (e.g., family and peer relationships) that must be taken into account for a comprehensive investigation.

Consistent with Kaufman et al.^18^, changes in one system (e.g., the relationship between parents and children) can alter the correlates affecting other systems (e.g., adolescents’ self-regulation). This study suggests that improvement in parent–child relationships can influence children’s behavior (i.e., participation in online activities), emotion (i.e., CER), and cognition (i.e., OSE^24,25^). Teenagers benefit from an emotionally secure family environment^55,56^. A friendly family relationship builds an environment that is more open to discussion among family members, and this kind of family system is likely to be more resilient and to be able to adapt to changes. Therefore, adolescents from such families can allocate more of their cognitive load to focus more effectively on their studies and daily life.

The current study has several limitations that could be addressed in future studies. First, the parents reported the parent–child relationship and parental monitoring, but their children may have different perceptions of these two parenting variables. Future studies could explore the effect of the perceived differences between parents and children in terms of the parenting variables. Second, our cross-sectional study may not reflect the long-term changes in adolescents’ well-being. A longitudinal study of the potential risk or protective factors that can affect adolescents’ well-being could be conducted to investigate the long-term changes in adolescents’ self-regulatory behavior, emotions, and cognition resulting from the pandemic, through the post-pandemic period, and into the New Normal. Third, this study did not take into account teacher-level or school-level factors. We cannot assume that all parents are naturally skilled at parent–child relationship management and parental monitoring. Teachers should provide professional support to educate parents on this theme, especially in a digital context. Therefore, from a multi-level perspective, a future study could include school-related factors (e.g., family–school cooperation in the digital realm), in addition to parent-related factors, to provide more insights into ways of supporting adolescents’ well-being during school suspension and after its resumption.

## Methods

### Context

This study was part of Hong Kong’s “eCitizen Education 360” project. It involved five comprehensive surveys of students, parents, teachers, school leaders, and ICT (information and communications technology) coordinators. Its aim was to prepare students for the changes in study and daily life in the new normal and to comprehensively enhance our community’s ability to improve educational opportunities, digital competence, and the well-being of students.

### Data collection procedure

All of the primary and secondary schools in all of Hong Kong’s 18 districts were invited to participate in this study on a voluntary basis through our project website (https://www.ecitizen.hk/360/call-for-participation). A person in charge (PIC) from each participating school notified us of their intention to take part. The specific grade levels and numbers of classes participating were decided by the individual schools based on their needs and administrative capacity. The links to the surveys for different roles were sent to the PIC, who helped to distribute the surveys to the corresponding participants for completion. Students took part in the survey with the assistance of their teachers.

Consent was obtained from the parents of participating students. Ethics approval for the study was obtained from the authors’ institution (the University of Hong Kong) before data were collected. The participants gave written informed consent to complete the survey and participate in the study.

All of the surveys were conducted anonymously through an online survey system, *Qualtrics*, from June 8, 2020 in secondary schools and from June 20, 2020 in primary schools. Respondents could choose to respond to the English or Chinese version of the survey. The Chinese version was translated from the original English version and was validated by back-translation. Data collected up to July 14 were included in our analysis, which excluded primary school students. About 550 school leaders, 790 teachers, 1300 parents, and 6300 students participated in the project.

### Samples

In this study, our sample consisted of 932 students and their parents from 23 secondary schools. Over 66% of the students were from junior secondary schools, and the rest were from senior secondary schools. The female students had a mean age of 14.80 years (SD = 1.56), and the male students had a mean age of 15.00 years (SD = 1.60). The parents had a diverse range of educational attainment (see Table 3).

**Table 3:** Summary of demographic information of participants.

		*N*	%
**Student**			
Gender			
	Male	265	28.43
	Female	666	71.46
	Missing	1	0.11
Grade			
	Junior secondary school	618	66.31
	Senior secondary school	313	33.58
	Missing	1	0.11
**Parent**			
Gender			
	Male	217	23.28
	Female	711	76.29
	Missing	4	0.43
Educational level			
	Junior secondary	204	21.89
	Senior secondary	316	33.91
	Associate degree	94	1.09
	Bachelor’s degree	199	21.35
	Master’s degree	115	12.34
	Missing	4	0.43

### Measures

#### Student surveys

The OSE survey was adapted from the Self-Efficacy Questionnaire for Children^57^. The survey consisted of six items that measured students’ beliefs in their capacity for online learning during the school suspension (i.e., “I could focus on my studies when there were other interesting things to do.”) scored on a 5-point Likert scale (1 = strongly disagree, 5 = strongly agree).

The WOR survey consisted of six items designed to measure students’ concerns about their studies and life after school resumption (e.g., “I cannot catch up with my schoolwork.”) scored on a 5-point Likert scale (1 = strongly disagree, 5 = strongly agree).

The CER survey consisted of three items adapted from the cognitive emotion regulation questionnaire^10^. The items measured the extent to which students were able to use cognitive strategies to cope with unpleasant events during school suspension (e.g., “I thought that I must accept it.”) scored on a 5-point Likert scale (1 = never, 5 = always).

The DSE survey consisted of five items that measured the extent to which a student participated in online socialization and entertainment activities daily during school suspension (e.g., chatting with friends via WhatsApp) scored on a 5-point scale (1 = not at all, 5 = more than 5 times a day).

The CyI survey was adapted from a validated instrument^58^ and measured students’ involvement as a perpetrator of cyberbullying, a victim, and a bystander. The participants reported their experiences of six cyberbullying events (e.g., something embarrassing or mean about another person) based on their roles in these events (i.e., bully, victim, both bully and victim, bystander, or never experienced).

#### Parent surveys

The PCR survey evaluated the parent’s relationship with their child during school suspension using four items (e.g., “I have understood my child’s ability more.”) scored on a 5-point Likert scale (1 = strongly disagree, 5 = strongly agree).

The PM survey consisted of three items that measured the extent to which parents monitored their children’s online behavior (e.g., “I monitored my child’s apps/websites/YouTube channels.”) scored on a 5-point Likert scale (1 = never, 5 = always).

### Analytic plan

#### Examining latent constructs and reliability

The latent construct of each factor, except for items that measured students’ CyI, was examined by applying a CFA using robust maximum likelihood estimation. The responses of these scales had acceptable ranges for normal distribution, with skewness ranging between −1 and 2 and kurtosis ranging between −2 and 2^59^. All of the CFA models were identified by setting the latent factor mean to 0 and the latent factor variances to 1. All of the item intercepts, item factor loadings, and item residual variances were freely estimated. The goodness of model fit was assessed by CFI, RMSEA, and SRMR. A CFI greater than .90 and RMSEA and SRMR values of less than .08 were considered as satisfying the model fit^60^. McDonald’s omega^61^ was used to calculate the reliability for each CFA model.

The general expression of reliability Omega is shown as follows:

1 $$\omega = \frac{{\left( {\mathop {\sum}\nolimits_1^I {\lambda _i} } \right)^2}}{{\left( {\mathop {\sum }\nolimits_1^I \lambda _i} \right)^2 + {\sum} {\theta _{ii} + 2{\sum} {\theta _{ij}} } }}$$

Where, *λ*_*i*_ represents the factor loading for Item *i*, *θ*_*ii*_ and *θ*_*ij*_ represent the error variance and error covariance, respectively. As a result, coefficient omega represents the proportion of variance in the observed total score attributable to all “modeled” sources of common variance. The coefficient omega ranges from 0 to 1, and a higher value indicates higher reliability of the scale. The coefficient omega listed above assumes uni-dimensionality, therefore, it is suitable for the single-factor model. The Omega hierarchical was adopted to estimate the reliability of the higher-order factor in the higher-order model in the current study^62^.

As there is no clear consensus on different categories of cyberbullying experience, this study used the nominal response model (NRM^63^) to measure the extent of CyI. The NRM is a flexible polytomous Item Response Theory model that does not require the ordering of item responses. For example, it does not assume the order of each category of cyberbullying experience before the estimation, but it estimates the order of each category based on the data. The current NRM assumes that a continuous latent trait (θ) influences unordered item responses, which is identified by setting the variance of θ equal to 1 and the mean of θ equal to 0, and then determines the estimate by maximum marginal likelihood using the Expectation-Maximization algorithm^64^. The Expected A-Posteriori method^65^ was adopted to compute factor scores for each participant, with a higher score indicating the participant had greater involvement in cyberbullying.

The nominal response model^63^ assumes a continuous latent factor (θ) accounts for the covariances among items without assuming the orders (i.e., nominal responses). Assume X_i_ is the response on the i^th^ item and p(X_i_ = *k*|*θ*) is the conditional probability of selecting response category k (k = 1,…,m_i_) for item i. the categories are nomial, meaning no assumption on selecting category 2 reflects a higher level of θ than selecting category 1. The NRM could be expressed as

2 $${{{\mathrm{P}}}}\left( {{{{\mathrm{X}}}}_{{{\mathrm{i}}}} = k{{{\mathrm{|}}}}\theta } \right) = \frac{{e^{a_{ik}\theta + c_{ik}}}}{{\mathop {\sum }\nolimits_{j = 1}^{m_i} e^{a_{ij}\theta + c_{ij}}}},$$

Where a_ik_ and c_ik_ are the category slope and category intercept parameters for the kth category of item I, respectively (see Table 4). In NRM, the probability that a person with trait-level θ chooses option k on item the expression on the right gives me, the ratio of selecting one category over the sum of all the other categories, as a function of θ with a varying category slope parameter (a_ik_) and a varying category intercept parameters c_ik_ for each of the m_i_ response categories. To identify the model, the mean and variance of θ are fixed to 0 and 1, respectively. Within each item, the order for the response categories with respect to the latent trait is determined by the value of a_ik_, a larger value of a_ik_ indicates a higher level of θ. For example, if a_ik_ > *a*_*iq*_, thus response k indicates higher θ than response q. The intercept parameter, c_ik_ reflect the relative frequency of selecting category k, where larger c_ik_ reflects relatively higher frequency for category k. After fitting the NRM using Eq. 3, item parameters and person’s latent trait could be estimated^66^. The empirical reliability of the cyberbullying involvement scale was estimated in the following way.

**Table 4:** Estimated Parameters of NRM.

	a1	a2	a3	a4	a5	c1	c2	c3	c4	c5
Item1	−28.72	5.64	6.42	6.16	10.51	−69.97	14.84	15.53	17.06	22.54
Item2	−2.75	0.07	−0.52	−0.28	3.48	−5.65	−0.22	−1.65	0.97	6.54
Item3	−20.18	2.38	4.19	4.43	9.18	−49.91	7.81	10.66	13.23	18.22
Item4	−17.03	−0.68	4.11	3.70	9.90	−37.83	−0.01	9.07	10.38	18.39
Item5	−21.46	3.11	4.47	4.32	9.56	−50.28	8.42	10.19	12.29	19.37
Item6	−13.71	0.99	2.90	2.79	7.03	−29.51	2.88	5.58	7.63	13.42

Category 1 to 5 are bully others, being bullied, both bully and being bullied, bystander, and no experiences, respectively. Higher a1 to a5 values indicate the higher level of each cyberbullying involvement of each category; Higher c1 to c5 values indicate the higher frequency of cyberbullying experience of each category.

From the NRM model in Eq. 3, we obtained the individual’s level of cyberbullying involvement (θ) and the standard error (SE(θ)) using the EAP method. Then, the empirical reliability ${\uprho}_{{{{\mathrm{xx}}}}^\prime }$ was calculated as the ratio of the true score variance and the total score variance, the sum of the true score variance and error variance^67^.

3 $${\uprho}_{{{{\mathrm{xx}}}}^\prime } = \frac{{\widehat {var}(\hat \theta )}}{{\widehat {var}\left( {\hat \theta } \right) + \widehat {SE}\left( {\hat \theta } \right)^2}}$$

Where, $\widehat {SE}\left( {\hat \theta } \right)^2$was obtained by averaging across the N standard errors of respective $\widehat {\theta _i}$ and squaring it.

**Fig. 4:**
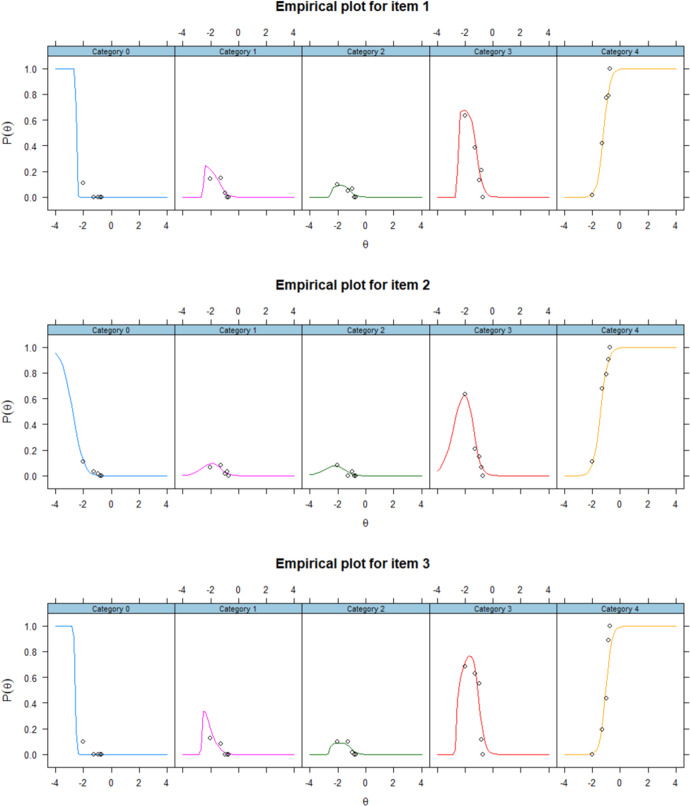

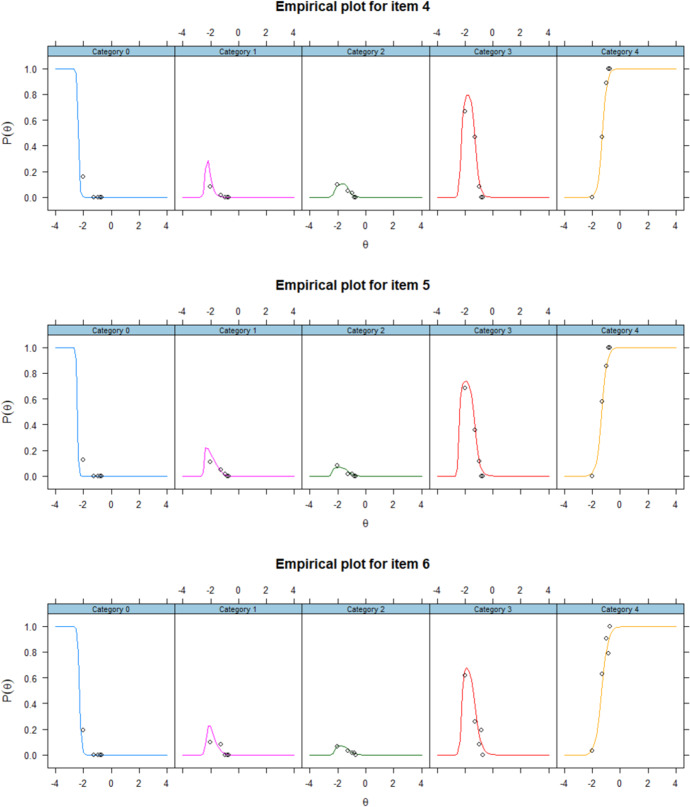
Empirical plots of NRM. The observed data (the dots) and predicted values (the lines) from NRM for all items.The small gap between the observed and predicted values indicates a good model-data fit.

Item fit of NRM was examined by viewing the residual plots of each plot. The difference between the line (model implied) and dots (data implied) represents the residuals. The smaller the residuals are, the better model-data fit is. The figures below presented the Empirical plots of all items of NRM. As shown in the Fig. 4 from item 1 to 6, the residuals were small enough to support achieving a good model fit^63^.

#### Exploring the relationships among parent and student factors

MG-SEM was performed using Mplus (version 8.1) to address the direct effects, indirect effects, and corresponding differences between male and female students. Bias-corrected confidence intervals with 10,000 bootstrapped samples were used to test the indirect effects. The measurement invariance between the sexes was examined and the partial measurement invariance was also determined. The structural relationships between the factors in the four groups were uncontaminated by measurement errors or measurement differences^68^. The male group was set as the reference group by fixing the factor means and variance to 0 and 1 respectively, to allow the estimation of female factor means and variances.

A standard stepwise procedure of testing measurement invariance between two gender groups was followed, in which the measurement invariance test started from a configural invariant model, a metric invariant model, and a scale invariant model.

Step 0. Test the CFA model in the whole, male, and female samples, respectively.

Step 1. A configural invariant model was specified. The CFA model for two gender groups was estimated simultaneously. All factor variances were fixed to 1, and factor means were fixed to 0 to set the scale. All loadings, residual variances, intercepts, and factor covariances were freely estimated. The model fit indices were checked to ensure the acceptable model fit was achieved.

Step 2. A metric invariant model was examined by constraining the items to have equal loadings across time. The factor variance was fixed to 1 in the male group but was freely estimated in the female group. All factor means were fixed to 0. Other parameters were constrained as the configural model. Then a Likelihood Ratio Test (LRT) was conducted to test if the metric invariant model did not fit worse than the configural model.

Step 3. A scalar invariant model was examined by constraining the same items had to have equal intercepts across genders; the factor means in the male group was fixed at 0, but other factor means were freely estimated. Other parameters constraints were set the same as the previous model. Then the LRT was conducted to test if the scalar invariant model did not fit worse than the metric model.

A similar procedure was conducted for each factor. In the empirical studies, when the full invariant model cannot be achieved, previous studies showed that a partial invariant model (a partial factor loading and partial intercept invariant model) could ensure the meaningful comparison of latent factor means^68^. Therefore, when the full invariant model was not achieved, we freely estimated some items across the group based on the modification indices and a content review. As a result, we freely estimated one item of measuring students’ digital socialization.

### Reporting summary

Further information on research design is available in the Nature Research Reporting Summary linked to this article.

## Supplementary information

Reporting Summary

## Data Availability

Data analyzed in the project are available upon request. Please contact the corresponding author.

## Code availability

The code for data analysis used in the project is available upon request. Please contact the corresponding author.
