# Supplementary material for: Understanding protective and risk factors affecting adolescents’ well-being during the COVID-19 pandemic
Source: NPJ Sci Learn. 2022 Dec 14;7:32. doi: 10.1038/s41539-022-00149-4 (PMC9748907; doi:10.1038/s41539-022-00149-4)
Supplement: Supplementary file 1 — Reporting Summary [file 41539_2022_149_MOESM1_ESM.pdf]

nature portfolio

Corresponding author(s): Cheng Yong Tan

Last updated by author(s): Jul 6, 2022

Reporting Summary

Nature Portfolio wishes to improve the reproducibility of the work that we publish. This form provides structure for consistency and transparency in reporting. For further information on Nature Portfolio policies, see our Editorial Policies and the Editorial Policy Checklist.

Statistics

For all statistical analyses, confirm that the following items are present in the figure legend, table legend, main text, or Methods section.

n/a	Confirmed	
☒	☐	The exact sample size (*n*) for each experimental group/condition, given as a discrete number and unit of measurement
☒	☐	A statement on whether measurements were taken from distinct samples or whether the same sample was measured repeatedly
☒	☐	The statistical test(s) used AND whether they are one- or two-sided<br>*Only common tests should be described solely by name; describe more complex techniques in the Methods section.*
☐	☒	A description of all covariates tested
☒	☐	A description of any assumptions or corrections, such as tests of normality and adjustment for multiple comparisons
☐	☒	A full description of the statistical parameters including central tendency (e.g. means) or other basic estimates (e.g. regression coefficient) AND variation (e.g. standard deviation) or associated estimates of uncertainty (e.g. confidence intervals)
☒	☐	For null hypothesis testing, the test statistic (e.g. F, t, r) with confidence intervals, effect sizes, degrees of freedom and P value noted<br>*Give P values as exact values whenever suitable.*
☒	☐	For Bayesian analysis, information on the choice of priors and Markov chain Monte Carlo settings
☐	☒	For hierarchical and complex designs, identification of the appropriate level for tests and full reporting of outcomes
☐	☒	Estimates of effect sizes (e.g. Cohen's *d*, Pearson's *r*), indicating how they were calculated

Our web collection on statistics for biologists contains articles on many of the points above.

Software and code

Policy information about availability of computer code

Data collection	Survey data was collected via Qualtrics.
Data analysis	Data was analyzed by using R and Mplus.

For manuscripts utilizing custom algorithms or software that are central to the research but not yet described in published literature, software must be made available to editors and reviewers. We strongly encourage code deposition in a community repository (e.g. GitHub). See the Nature Portfolio guidelines for submitting code & software for further information.

Data

Policy information about availability of data

All manuscripts must include a data availability statement. This statement should provide the following information, where applicable:

- Accession codes, unique identifiers, or web links for publicly available datasets
- A description of any restrictions on data availability
- For clinical datasets or third party data, please ensure that the statement adheres to our policy

The original contributions presented in this study are included in the article/supplementary material, further inquiries can be directed to the corresponding author/s.

Human research participants

Policy information about studies involving human research participants and Sex and Gender in Research.

Reporting on sex and gender	Student data included 265 (28.43%) males, 666 (71.46%) females and 1 (0.11%) missing. Parent data included 217 (23.28%) males, 711 (76.29%) females, and 4 (0.43%) missing in gender
Population characteristics	In this study, the sample comprised 932 students and their parents from 23 secondary schools. Over 66% of the students were from junior secondary schools, and the rest were from senior secondary schools. In the secondary school, female students had a mean age of 14.80 years (SD = 1.56), and male students had a mean age of 15.00 (SD=1.60).
Recruitment	This study is a part of a project entitled "The eCitizen Education 360" (e360 for short), all primary and secondary schools in Hong Kong were invited and then they participated on a voluntary basis. Surveys were administered during June-July 2020 after four months of the first wave of school suspension in Hong Kong.
Ethics oversight	Consent was obtained from the parents of participating students. Ethics approval for the study was obtained from the authors' institution (The University of Hong Kong) before data were collected.

Note that full information on the approval of the study protocol must also be provided in the manuscript.

Field-specific reporting

Please select the one below that is the best fit for your research. If you are not sure, read the appropriate sections before making your selection.

☐ Life sciences ☒ Behavioural & social sciences ☐ Ecological, evolutionary & environmental sciences

For a reference copy of the document with all sections, see nature.com/documents/nr-reporting-summary-flat.pdf

Behavioural & social sciences study design

All studies must disclose on these points even when the disclosure is negative.

Study description	It is a quantitative study, which aims to enhances capacities of both individual student, families, and the education community to mitigate the negative impacts posed by this global pandemic as well as improve the abilities of students, families and education communities to cope with future challenges.
Research sample	Secondary school students and their parents in Hong Kong, SAR.
Sampling strategy	All primary and secondary schools in Hong Kong were invited and then they participated on a voluntary basis.
Data collection	Data was collected online via Qualtrics. All primary and secondary schools in Hong Kong were invited and then they participated on a voluntary basis.
Timing	June to July 2020.
Data exclusions	NA
Non-participation	We collected 5216 secondary school students and 1613 parents; however, we were able to match 932 parent-child data.
Randomization	NA

Reporting for specific materials, systems and methods

We require information from authors about some types of materials, experimental systems and methods used in many studies. Here, indicate whether each material, system or method listed is relevant to your study. If you are not sure if a list item applies to your research, read the appropriate section before selecting a response.

Materials & experimental systems

n/a	Involved in the study	
☒	☐	Antibodies
☒	☐	Eukaryotic cell lines
☒	☐	Palaeontology and archaeology
☒	☐	Animals and other organisms
☒	☐	Clinical data
☒	☐	Dual use research of concern

Methods

n/a	Involved in the study	
☒	☐	ChIP-seq
☒	☐	Flow cytometry
☒	☐	MRI-based neuroimaging
